# Beneficial Effects of Robot-Assisted Gait Training on Functional Recovery in Women after Stroke: A Cohort Study

**DOI:** 10.3390/medicina57111200

**Published:** 2021-11-03

**Authors:** Nicola Lamberti, Fabio Manfredini, Luc Oscar Lissom, Susanna Lavezzi, Nino Basaglia, Sofia Straudi

**Affiliations:** 1 Department of Neuroscience and Rehabilitation, University of Ferrara, 44121 Ferrara, Italy; fabio.manfredini@unife.it (F.M.); nino.basaglia@unife.it (N.B.); sofia.straudi@unife.it (S.S.); 2 Unit of Rehabilitation Medicine, University Hospital of Ferrara, 44124 Ferrara, Italy; s.lavezzi@ospfe.it; 3 Doctoral Program in Translational Neurosciences and Neurotechnologies, University of Ferrara, 44121 Ferrara, Italy; lucoscar.lissom@unife.it

**Keywords:** stroke, multidisciplinary rehabilitation, robot-assisted gait training, walking, gender, exercise prescription, women’s health

## Abstract

*Background and Objectives:* Robot-assisted gait training (RAGT) could be a rehabilitation option for patients after experiencing a stroke. This study aims to determine the sex-related response to robot-assisted gait training in a cohort of subacute stroke patients considering mixed results previously reported. *Materials and Methods:* In this study, 236 participants (145 males, 91 females) were admitted to a rehabilitation facility after experiencing a stroke and performed RAGT within a multidisciplinary rehabilitation program. Functional Independence Measure (FIM) and Functional Ambulatory Category (FAC) were assessed at admission and discharge to determine sex-related outcomes. *Results:* At the baseline, no significant difference among sexes was observed. At the end of rehabilitation, both males and females exhibited significant improvements in FIM (71% of males and 80% of females reaching the MCID cut-off value) and FAC (∆score: men 1.9 ± 1.0; women 2.1 ± 1.1). A more remarkable improvement was observed in women of the whole population during the study, but statistical significance was not reached. When analysing the FAC variations with respect to the total number of RAGT sessions, a more significant improvement was observed in women than men (*p* = 0.025). *Conclusion:* In conclusion, among subacute stroke patients, benefits were observed following RAGT during a multidisciplinary rehabilitation program in both sexes. A greater significant recovery for women with an ischemic stroke or concerning the number of sessions attended was also highlighted. The use of gait robotics for female patients may favour a selective functional recovery after stroke.

## 1. Introduction

As a leading cause of death and long-term disability [1], stroke poses a significant danger to women. After a stroke, women showed a greater death rate attributed to stroke or other cardiovascular diseases [2], higher disability, and a poorer quality of life compared to males [3]. Similarly, in the presence of conflicting data on functional recovery [4,5], more frequent presence of physical impairments and limitations in activities of daily living [5], depression [6], fatigue [7], and worse cognitive outcomes were reported in women [4,5].

In stroke survivors, mobility is often limited by walking impairment [8,9] and restoration of walking ability by gait rehabilitation is a relevant objective of the recovery process [10]. In recent years, the introduction of robotics [11,12] showed interesting rehabilitation outcomes for stroke survivors [13,14], offering an alternative to conventional rehabilitation [15]. Robotic rehabilitation provides intensive, task-oriented, repeated [16,17], and personalised work for a favourable recovery and the plasticity-dependent response of stroke survivors [18,19], both after an ischemic or haemorrhagic event [20]. In addition, the robotic system, allowing partial or total body weight bearing, enables the enrolment of non-ambulatory patients and the prevention of falls during training sessions [21]. In chronic stroke patients (as for other neurologic diseases [22]), studies failed to demonstrate a more favourable impact of electromechanically or technology-assisted gait training in recovery compared to conventional overground gait training [23,24]—or its effectiveness in combination with physiotherapy to achieve independent walking [12,25]. However, the effectiveness of robotic training was reported for non-ambulatory patients rather than for ambulatory stroke survivors [12,18], suggesting that this approach might represent a rehabilitative strategy for targeted populations.

Rehabilitation is a critical issue for women, considering their lower response or adherence to rehabilitation for various chronic diseases [26,27,28,29,30,31]. After a stroke, sex was associated with lower post-stroke physical activity [32] and different improvements in physical function following home-based rehabilitation among home-dwelling patients [33]. So far, even though differences in functional outcomes after inpatient rehabilitation have been found between sexes [34,35], no evidence is available on high-intensity gait rehabilitation after suffering a stroke. A randomised trial comparing robot-assisted gait training (RAGT) versus conventional training in a population of neurologic patients, including stroke survivors, showed that walking recovery in the RAGT group was significantly improved among females compared with males [36]. However, inclusion criteria for patients enrolled in a clinical trial are often very narrow, and patients with comorbidities or severe motor function are usually excluded. For these reasons, the investigation of sex differences in a larger, ecological, clinical practice-based population of patients affected by stroke is warranted to report the responses to RAGT rehabilitation on ambulatory capacity. This study aims to compare the benefits between males and females in terms of functional recovery obtained after RAGT during a multidisciplinary rehabilitation program in a cohort of subacute stroke patients. If differences in outcomes are observed, considering the previously known sex differences in occurrence, severity of stroke, and therapies [37], gender-specific rehabilitative treatment might be considered.

## 2. Materials and Methods

We retrospectively analysed a prospectively collected dataset of patients with subacute stroke that received inpatient multidisciplinary rehabilitation at the Department of Physical and Rehabilitation Medicine at the University Hospital of Ferrara, Italy. The ethics committee CE-AVEC approved the study, but written informed consent was not collectable from all patients because some of them were no longer attending the rehabilitation clinics.

### 2.1. Subjects

Subacute stroke patients that underwent a multidisciplinary rehabilitation program between May 2007 and April 2018 were studied.

Inclusion criteria were: male and female patients aged > 18 years; ischemic or haemorrhagic stroke onset within 90 days from rehabilitation admission; Functional Ambulatory Category (FAC) and Functional Independence Measure (FIM) at entry ≤3 and ≤90, respectively.

Exclusion criteria were: impossibility to perform RAGT due to medical instability (cardiorespiratory conditions preventing exercise therapy, e.g., unstable angina, severe heart failure, etc.), severe cognitive impairments (mini-mental state examination score <18/30), severe lower limb spasticity, or skin lesions.

### 2.2. Interventions

All patients during the hospital stay underwent RAGT using the Lokomat treadmill (Hocoma AG, Volketswil, Switzerland). During these sessions, subjects wore a harness attached to a system to provide bodyweight support, and they walked on the treadmill with the help of a robotic-driven gait orthosis. The patient’s legs were guided according to a physiological gait pattern with the possibility to adjust the torque of the knee and hip drives. Each training session lasted for an hour with 30 min of real walking time due to the setup time. During the first treatment session, the parameters were set according to the patient’s functional characteristics. However, for the patients included in the study, a 50% relief of body weight and 100% assistance provided by the robot were scheduled [38]. Treadmill speed, bodyweight support, and guidance force were progressively adjusted during training progression. The training lasted for a minimum of seven sessions, with variable frequencies (1–5 times/week).

Concomitantly, all patients benefited from an intensive, multidisciplinary rehabilitation program tailored to each individual’s needs (conventional motor rehabilitation, occupational therapy, speech, and cognitive therapy for a maximum of 6 h/day and at least 3 h/day for 5 days a week.) After admission, each patient was evaluated by a rehabilitation team that defined a specific program according to the WHO International Classification of Function framework [39]. Discharge was decided after a teamwide clinical evaluation of the patient’s functional status was performed, in addition to considering the patient’s expected recovery.

For the rehabilitation program, physiatrists, physiotherapists, speech therapists, and psychologists were involved during the entire hospital stay.

### 2.3. Outcome Parameters

A dataset containing information about patients’ demographics, including stroke characteristics, days from stroke to rehabilitation, length of hospital stay, and number of RAGT sessions, was compiled.

The primary outcome of the study was the FAC. As a secondary outcome, we considered the FIM, taking into account both its total score (FIMtot) and the motor (FIMmot) and cognitive (FIMcogn) domains. FIM efficiency (FIM gain/length of stay) was also calculated to monitor any sex-differences in cost efficiency. The FIM is an 18-item, clinician-reported scale that assesses function in six areas: self-care, continence, mobility, transfers, communication, and cognition. Each item is rated on a seven-level classification scale by an experienced therapist [40]. For this study’s purpose, the minimal clinically important difference (MCID) for the FIM total score was 22, according to Beninato et al. [41].

The FAC is a functional walking test that evaluates ambulation ability using a six-point scale by determining how much human support the patient requires when walking [42].

Both scales were collected at admission to the rehabilitation facility and discharge. Variations of both FAC and FIM were also normalised to the total number of RAGT sessions performed.

### 2.4. Statistical Analysis

The data distribution was verified through the Shapiro–Wilk test. The baseline comparison between the two groups (male and female) was obtained through a chi-square test for categorical variables (ischemic or haemorrhagic stroke) or the Mann–Whitney test for continuous variables.

For both primary and secondary outcomes (FIM and FAC), within-group comparisons were performed via Wilcoxon tests. Between-group comparisons for all outcomes were obtained using Mann–Whitney tests due to nonnormal data distribution. For all outcomes, Kruskal–Wallis tests were conducted to determine gender differences according to stroke type (ischemic or haemorrhagic).

A *p*-value < 0.05 was considered statistically significant. Data analyses were performed with MedCalc statistical software, version 19.2 (MedCalc Software Ltd., Ostend, Belgium).

## 3. Results

**Figure 1:**
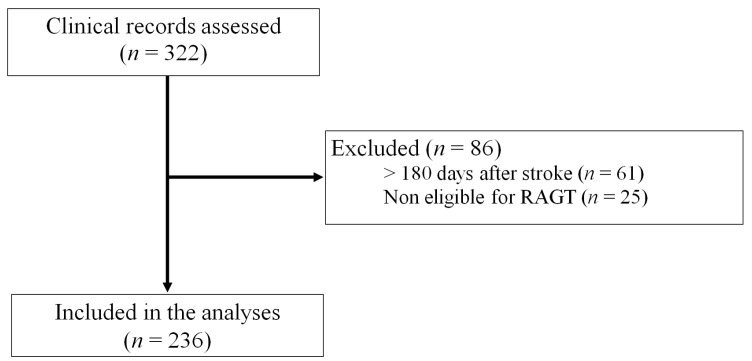
Flow diagram of participants.

Three hundred and twenty-two stroke patients admitted in the rehabilitation clinics from 2007 to 2018 who received RAGT were assessed for eligibility. Eighty-six patients were excluded because they did not match the inclusion criteria (Figure 1). Within the analysed sample of patients, 91 were females (39%) and 145 were males (61%).

### 3.1. Clinical and Demographic Characteristics at the Admission of Rehabilitation

At admission, the two groups were not different in terms of demographics nor clinical characteristics. Moreover, the outcome measures of FIM and FAC were balanced between men and women. Data are reported in Table 1.

### 3.2. Differences in Rehabilitation Treatments

Both groups showed a comparable length of hospital stay, calculated at 102 ± 56 days for females and 108 ± 60 days for males (*p* = 0.41). According to our Rehabilitation Department’s standard of care, both groups of patients underwent conventional physiotherapy sessions at least 3 h per day, Monday through Friday. In addition, a similar number of RAGT sessions were executed, with a mean of 14 ± 8 for females compared to 15 ± 8 for males (*p* = 0.22). No differences in RAGT duration were found (females 30 ± 3 vs. males 30 ± 4 min).

Finally, both groups showed the same RAGT frequency per week of 2 ± 1 sessions (*p* = 0.65), suggesting that a comparable amount of RAGT rehabilitation was given to both groups of patients.

### 3.3. Comparison of Outcomes

Both groups significantly improved on all the mobility scales considered in the study (Table 2). No between-group differences were observed for all outcomes, except for women that exhibited a higher, although not significant, FAC variation (2.1 ± 0.7 versus 1.9 ± 0.8 respectively; *p* = 0.11).

This finding was confirmed by analysing the ratio between the FAC variation and the number of RAGT sessions, which showed a significantly greater improvement in women (0.20 ± 0.16) compared to men (0.16 ± 0.14) (Table 3).

When considering the number of patients who reached the MCID for the FIM scale, no sex differences were observed, with 71% of men and 80% of women reaching the cut-off value (*p* = 0.15).

FIM efficiency was slightly higher in women (0.47 ± 0.44) compared with men (0.42 ± 0.31) without any differences (*p* = 0.26).

Finally, at the end of rehabilitation, 28 (19%) males and 17 females (19%) reached gait independence (defined as FAC ≥ 4), again without any between-group difference (*p* = 0.90).

### 3.4. Sex Differences According to the Type of Stroke

Women affected by an ischemic stroke showed significantly greater improvement in FIMtot and FAC compared to women that experienced a haemorrhagic stroke. The former subgroup also showed a statistically significant difference for both scales regarding men affected by an ischemic stroke.

**Figure 2:**
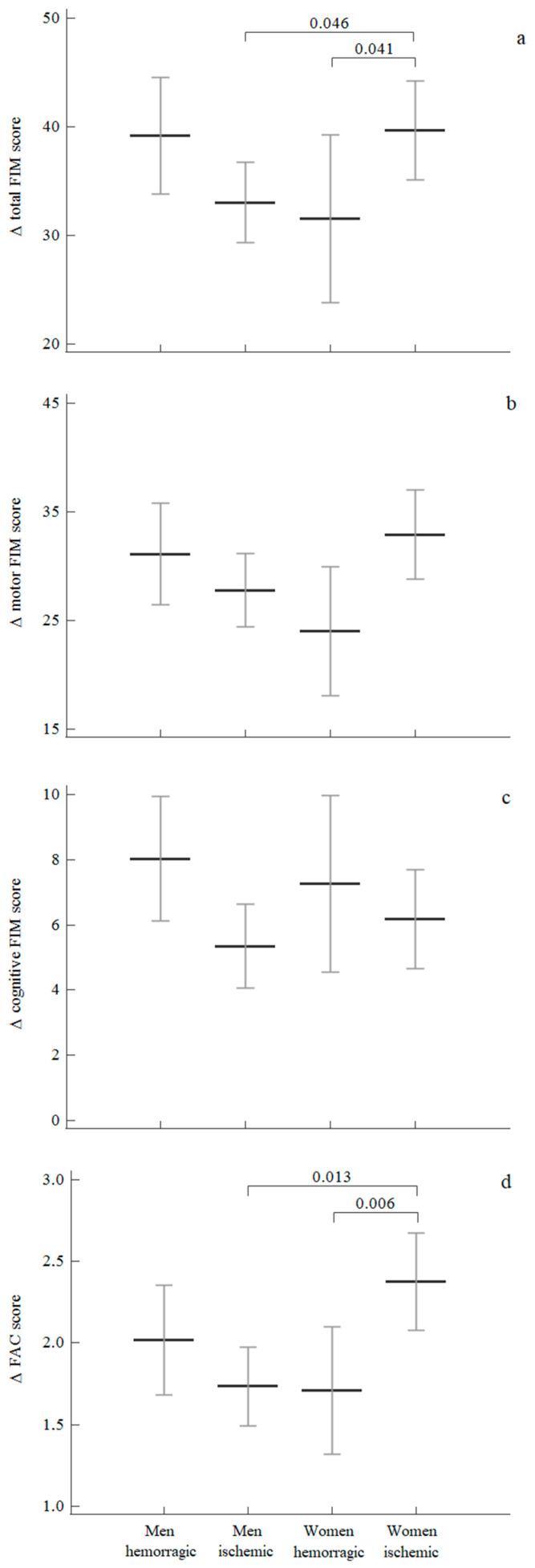
Mean and 95% confidence interval for (**a**) FIMtotal, (**b**) FIMmotor, (**c**) FIMcognitive and (**d**) FAC variations after rehabilitation in the two groups categorised by stroke type. Legend: Between-group comparison performed through Kruskal–Wallis tests.

No differences were observed in men according to stroke type (Figure 2).

## 4. Discussion

This 10-year, single-centre retrospective study carried out in a large population of subacute stroke survivors admitted to a rehabilitation facility highlighted a favourable response to robot-assisted gait training without any significant sex difference. However, a better effect of RAGT in women affected by ischemic strokes was observed.

The study offers several points of discussion to be addressed. 

Scientific literature reports conflicting findings concerning functional and quality of life recovery for female stroke survivors. In particular, several papers reported the worst outcome for women after rehabilitation [4,43,44,45], while others observed similar or better improvements for females than males [46,47]. To the best of our knowledge, this is the largest pragmatic cohort study based on clinical care exploring sex differences among subacute stroke survivors who received RAGT during their rehabilitation stay. Women represented 39% of our cohort, which was a superimposable value compared with the ARTIC study [38]. Our study also confirmed similar results between the two sexes for FAC and all FIM scores. In this regard, it is noteworthy that in our study, the total variation in FAC score was almost four times greater than the mean variation of 0.51 reported in a recent meta-analysis [25]. Several aspects may have influenced this finding, such as, a lower FAC level at the baseline in our population, the different number of RAGT sessions completed in the different trials, or simply the fact that in our study, the FAC variation was determined at the admission and discharge from a rehabilitation unit, instead of immediately before and after RAGT treatment.

Interestingly, when normalising the FAC variations for the number of RAGT sessions performed, women exhibited a significantly greater response to RAGT given an equal number of training sessions. This represents a relevant aspect, considering women’s limited adherence to rehabilitation in several chronic diseases with respect to men, as previously reported [26,27,28,29,30,31]. Moreover, this finding supported the greater responsiveness of women to RAGT, leading to the hypothesis that they need fewer sessions to achieve the same gait function.

Concerning robot-assisted gait training, in the 2016 AHA guidelines [48], RAGT achieved an IIb class of recommendation with an A level of evidence to improve motor function and mobility after stroke in combination with conventional therapy. In our study, RAGT proved to be effective with 75% of subacute stroke patients that reached the minimal clinically important difference for FIM total score, and with 19% of patients that achieved independent ambulation, defined by a FAC score ≥ 4, confirming previous results reported in several literature reviews [18,25,49]. Moreover, for the number of subjects that reached the MCID, no sex differences were noted. As an additional element of interest, a greater functional recovery was observed in ischemic stroke-affected women compared to women affected by haemorrhagic stroke and men. This finding, confirmed for both FAC and FIMtot scales, clashes with the previous results in the literature, where haemorrhagic stroke had an equal [50] or better functional recovery [51,52]. However, our cohort, with a mean age of 62 years old, was significantly younger than the worldwide stroke population [53], showing that older women with multiple comorbidities, severe functional impairment, and a poorer outcome did not usually receive RAGT in clinical practice. This age-dependent difference can partially explain why women with ischemic stroke might have a more favourable outcome than men in our study [54].

Therefore, according to the significantly better outcomes observed in this study for women with ischemic stroke, RAGT may represent a beneficial rehabilitation option, especially for these patients.

This aspect opens another important field of discussion, as the rehabilitation in chronic stroke survivors ensures the maintenance of mobility and functional independence after the in-hospital phase. Both home-based and community-based interventions have proved their effectiveness [32,55,56,57], but sex differences need to be further investigated in this context.

The study presents several limitations. First, it is a retrospective study that encompassed a concomitant rehabilitation treatment and RAGT during the hospital stay; in addition, the outcome measures were collected only at entry and discharge, and objective measures of physical functioning (e.g., walk tests) were not reported. Data related to the anthropometric characteristics of patients were not gathered.

## 5. Conclusions

In conclusion, a conventional rehabilitation treatment empowered by RAGT promoted gait recovery in young stroke patients with severe functional impairment without sex differences. In the studied population, selective improvements were observed for young females after ischemic stroke, but further prospective studies are needed to investigate the issue of sex-selective improvements in this population.

## Figures and Tables

**Table 1:** Characteristics of the two groups of patients at hospital admission.

	Women (*n* = 91)	Men (*n* = 145)	Between-Group *p*
Age, years	64 (59–67)	64 (61–66)	0.74
Ischemic stroke, *n* (%)	56 (60)	90 (62)	0.68
Haemorrhagic stroke, *n* (%)	35 (40)	55 (38)	0.72
Days since stroke	35 (28–41)	35 (30–43)	0.69
FIM, total score	45 (35–51)	46 (41–50)	0.54
FIM, motor component	22 (19–25)	20 (19–22)	0.88
FIM, cognitive component	22 (19–25)	24 (20–27)	0.46
FAC	0.3 ± 0.7	0.4 ± 0.6	0.16

Legend: Data are reported as median and at a 95% confidence interval for continuous variables or number (percentage) for categorical variables. For FAC value, data are expressed as mean ± standard deviation. Abbreviations: FIM, functional independence measurement; FAC, functional ambulatory classification.

**Table 2:** Outcomes of the study for the two groups.

	Women (*n* = 91)		Men (*n* = 145)		Between-Group *p* in Variations Admission-Discharge
	Admission	Discharge	Admission	Discharge	
FIM, total	45 (35–51)	87 (79–92) **	46 (41–50)	83 (78–91) **	0.75
FIM, motor	22 (19–25)	56 (49–60) **	20 (19–22)	54 (50–61) **	0.75
FIM, cognitive	22 (19–25)	30 (29–31) **	24 (20–27)	31 (30–21) **	0.97
FAC	0.3 ± 0.7	2.4 ± 1.3 **	0.4 ± 0.6	2.3 ± 1.4 **	0.11

Abbreviations: FIM, Functional Independence Measurement; FAC, Functional Ambulatory Classification. Legend: Data are reported as median and at a 95% confidence interval. For FAC value, data are expressed as mean ± standard deviation. Within-group comparison by Wilcoxon tests: ** *p* < 0.01. Data are expressed as median (at a 95% confidence interval) or mean ± standard deviation.

**Table 3:** Variations of outcome measures normalised for the number of RAGT sessions in the two groups.

	Women (*n* = 91)	Men (*n* = 145)	*p*
∆FIM, total score	3.43 ± 2.87	2.89 ± 2.13	0.10
∆FIM, motor component	2.80 ± 2.38	2.41 ± 1.92	0.15
∆FIM, cognitive component	0.18 ± 0.27	0.14 ± 0.15	0.14
∆FAC	0.20 ± 0.16	0.16 ± 0.14	0.025

Abbreviations: FIM, Functional Independence Measurement; FAC, Functional Ambulatory Classification. Legend: Data are reported as mean ± standard deviation. Between-group comparison was performed through a Mann–Whitney test.

## Data Availability

Dataset is available upon request to the corresponding author.
